# Supplementary material for: Tensile strain and altered synovial tissue metabolism in human knee osteoarthritis
Source: Sci Rep. 2022 Oct 17;12:17367. doi: 10.1038/s41598-022-22459-8 (PMC9576717; doi:10.1038/s41598-022-22459-8)
Supplement: Supplementary file 1 — Supplementary Information 1. [file 41598_2022_22459_MOESM1_ESM.zip › 261620]

**Running head:** Tensile strain and synovial metabolism

**Tensile strain and altered synovial tissue metabolism in human knee osteoarthritis**

Holly T. Philpott, PhD^1,2^ (ORCID ID: 0000-0002-6146-0241); hphilpo2@uwo.ca

Trevor B. Birmingham, PT PhD^1,2^ (ORCID ID: 0000-0002-3300-7677); tbirming@uwo.ca

Benoit Fiset, BSc GrDip Bioinformatics^3^ (ORCID ID: 0000-0003-0372-8868); benoit.fiset@mcgill.ca

Logan A. Walsh, PhD^3,4^ (ORCID ID: 0000-0001-8771-2577); logan.walsh@mcgill.ca

Mitchell C. Coleman, PhD^5,6^ (ORCID ID: 0000-0002-5116-801X); mitchell-coleman@uiowa.edu

Cheryle A. Séguin, PhD^2,7^ (ORCID ID: 0000-0001-6503-9107); cheryle.seguin@schulich.uwo.ca

C. Thomas Appleton, MD PhD*^2,7,8^ (ORCID ID: 0000-0001-7148-0767); tom.appleton@sjhc.london.on.ca

**Affiliations:**

^1^Faculty of Health Sciences, Western University, London, ON, Canada, N6G 1H1

^2^Bone and Joint Institute, Western University, London, ON, Canada, N6A 5B5

^3^Rosalind and Morris Goodman Cancer Research Centre, McGill University, Montreal, Quebec, Canada, H3A 1A3

^4^Department of Human Genetics, McGill University, Montreal, Quebec, Canada, H3A 0C7

^5^Department of Orthopedics and Rehabilitation, University of Iowa, Iowa City, IA, USA, 52242

^6^Department of Radiation Oncology, University of Iowa, Iowa City, IA, USA, 52242

^7^Department of Physiology and Pharmacology, Schulich School of Medicine and Dentistry, Western University, London, ON, Canada, N6A 5C1

^8^Department of Medicine, Schulich School of Medicine and Dentistry, Western University, London, ON, Canada, N6A 5C1

***Corresponding Author:**

Tom Appleton MD PhD FRCPC

SJHC Rheumatology Centre

268 Grosvenor St., London, ON, Canada, N6A 4V2
t. (519) 646-6319 (clinic office)
r. (519) 661-3768 (research office)
e. [tom.appleton@sjhc.london.on.ca](mailto:tom.appleton@sjhc.london.on.ca)

**Word count:** 4,476

**Abstract**

*Objectives:* Synovium is critical for maintaining joint homeostasis and may contribute to mechanical responses during joint movement. We investigated mechanical responses of whole synovium from patients with late-stage knee osteoarthritis (OA).

*Methods:* Synovium samples were collected during total knee arthroplasty and assigned to histopathology or cyclic 10% tensile strain loading, including i) static (control); ii) low-frequency (0.3 Hz); and iii) high-frequency (1.0 Hz) for 30-minutes. After 6-hours incubation, tissues were bisected for RNA isolation and immunostaining (3-nitrotyrosine; 3-NT). RNA sequencing was analyzed for differentially expressed genes and pathway enrichment. Cytokines and lactate were measured in conditioned media.

*Results:* Compared to controls, low-frequency strain induced enrichment of pathways related to interferon response, Fc-receptor signaling, and cell metabolism. High-frequency strain induced enrichment of pathways related to NOD-like receptor signaling, high metabolic demand, and redox signaling/stress. Metabolic and redox cell stress was confirmed by increased release of lactate into conditioned media and increased 3-NT formation in the synovial lining.

*Conclusion:* Late-stage OA synovial tissue responses to tensile strain include frequency-dependent increases in inflammatory signaling, metabolism, and redox biology. Additional research should be completed to understand how synovial responses to mechanical loading can help or worsen synovial tissue oxidative stresses caused by OA.

**Key words:** synovium, mechanobiology, knee osteoarthritis

**INTRODUCTION**

The synovium is vital for joint homeostasis and experiences tensile and convective forces during flexion-extension joint movement including walking^1,2^. Synovial physiologic responses to mechanical loading may therefore help or worsen joint outcomes in osteoarthritis (OA). Although healthy synovium delivers nutrients and produces lubricating molecules^3^, OA induces marked changes in synovial tissues, which impair joint homeostasis and exacerbate pathogenesis^3,4^. Synovial inflammation predicts worse clinical outcomes in patients with knee OA^5-10^. Therefore, identifying the synovial biological mechanisms involved in OA pathogenesis is critical for developing new disease-modifying therapies for this debilitating disease.

Although a great deal is known about the effects of mechanical loading on cartilage little research exists on mechanical loading in synovial tissues. Mechanical stresses on articular cartilage invoke inflammatory, redox, and metabolic changes in cellular physiology, which influence OA pathogenesis^11^ and could similarly disrupt synovial function directly, and/or through crosstalk between cartilage and synovium. Recent descriptions of biological crosstalk between synovial cells and chondrocytes^12^ demonstrate the importance of considering synovial biology in addition to OA-driven cartilage degradation.

A paradoxical relationship exists between OA and physical activity. Where physical activity or exercise improves joint pain and function in the medium to long-term^13,14^ but many patients experience pain, stiffness, and joint inflammation during physical activity, and acute improvement with rest^15-17^. This suggests that differences exist in the mechanisms mediating the short-term, versus medium- to long-term effects of physical activity. Given recent discoveries underscoring the key relationship of the synovium to joint outcomes^5-10^, especially pain, we propose that OA synovium may display aberrant physiologic responses to mechanical stimuli, which in turn could contribute to OA pathogenesis, disease progression, and worse patient outcomes.

A recent study characterized the compressive properties and permeability of healthy synovium^18^. However, a major gap remains in our understanding of the mechanobiology of OA synovium. Importantly, tensile strain loading of synovial cells *in vitro* has been shown to induce changes in extracellular matrix, inflammatory, and oxidative stress gene expression^19-22^, all of which could impact joint health and homeostasis. Therefore, our objective was to assess the physiologic responses of intact human synovial tissues to mechanical loading with cyclic tensile strain. We investigated synovial tissue responses in the clinical context of patients with late-stage knee OA, who are most likely to have physical activity-related symptoms. Since macrophages are the most abundant immune cell type in OA synovium^23,24^, we also explored whether macrophage content in the synovial lining modifies the response to mechanical load.

**RESULTS**

Synovial tissue samples were from 10 patients undergoing TKA recruited consecutively from January 2019 to July 2019. For RNA sequencing, we included n=6 patients with synovial tissue RNA samples that met RNA quality/quantity requirements. Baseline demographics, clinical characteristics, and synovial histopathology for all 10 patients and the sub-group of 6 patients included for RNA sequencing are presented in Table 1.

| **Table 1**  **Patient demographics and clinical characteristics and synovial histopathology** | | |
| --- | --- | --- |
| **Characteristic** | **All patients (n =10)** | **RNA sequencing sub-group (n=6)** |
| **Age, years** | 67.4 ± 7.9 [54-77] | 66.3 ± 9.5 [54-77] |
| **Sex, n (%)**  Female  Male | 4 (40.0%)  6 (60.0%) | 2 (33.3%)  4 (66.7%) |
| **BMI, (kg/m^2^)** | 35.5 ± 6.1 [29.5-45.4] | 36.1 ± 5.6 [29.5-44.6] |
| **KL grade, n (%)**  Grade 3  Grade 4 | 3 (30.0%)  7 (70.0%) | 1 (16.7%)  5 (83.3%) |
| **KOOS Pain subscale, (0-100)** | 39.6 ± 22.2 [0-67] | 39.5 ± 24.5 [0-67] |
| **Histopathology, mean score**  Synovial Lining thickness  Sub-synovial infiltrate  Fibrin Deposition  Vascularization  Fibrosis  Perivascular edema | 0.7 ± 0.4 [0.4-1.6]  1.2 ± 0.7 [0.6-2.4]  0.8 ± 0.3 [0-1]  1.8 ± 0.9 [0-3.0]  1.3 ± 0.6 [0.6-2.5]  0.7 ± 0.6 [0-1.6] | 0.6 ± 0.4 [0.4-1.0]  1.3 ± 0.6 [0.6-2.2]  0.8 ± 0.4 [0-1]  1.7 ± 1.2 [0-3.0]  1.4 ± 0.5 [0.6-2.5]  0.8 ± 0.8 [0-1.6] |
| **Comorbidities, n (%)**  Hypertension  Dyslipidemia  Diabetes (Type II)  Obesity  Smoking  Active  Previous  Chronic liver disease  Chronic kidney disease | 8 (80.0%)  6 (60.0%)  4 (40.0%)  7 (70.0%)  0 (0.0%)  2 (20.0%)  0 (0.0%)  0 (0.0%) | 4 (66.7%)  3 (50.0%)  2 (33.3%)  5 (83.3%)  0 (0.0%)  1 (16.7%)  0 (0.0%)  0 (0.0%) |
| Mean ± SD (minimum, maximum) or n (%)  BMI, body mass index; KL, Kellgren-Lawrence grade; KOOS, Knee Injury and Osteoarthritis Outcome Score | | |

**OA synovial tissue transcriptomic responses to cyclic tensile strain**

*Significantly dysregulated genes*

We explored differential gene expression induced by cyclic tensile strain in synovial tissues (Figure 1). Compared to static control tissues, 1543 genes were significantly differentially expressed in OA synovial tissue after low-frequency tensile strain. Of these, 1463 genes decreased, 80 genes increased, and 1021 (66.2%) corresponded to non-coding genes. High-frequency strain induced differential expression of 101 genes, with 77 genes decreased, 24 genes increased, and 73 (72.3%) genes were non-coding. Only 27 genes were differentially expressed in both low- and high-frequency tensile strain conditions (Figure 1A).

Of the 522 coding genes that were differentially expressed following low-frequency tensile strain (vs. control), 467 coding genes decreased, and 55 genes increased (Figure 1B). Genes involved in circadian rhythm (*PASD1*), cell metabolism (*CALHM4*, *PKLR, G6PC2*), pain signaling (*TRPM8, HTR3A),* innate immunity *(REG1A, ASB18*), and the olfactory receptor gene family (*OR51A7, OR5R1, OR2T1*) were decreased, whereas genes involved in immune regulation (*CD14, CXCL10, TRBV2, DOK2, XCL1, ADAMDEC1, PTPRO, SLAMF8*) and cell metabolism (*MT1F, GBP1, GBP4*) were increased.

Of the 28 coding genes significantly differentially expressed following high-frequency tensile strain (vs. control), 19 genes decreased and 9 increased (Figure 1B). Genes involved in transmembrane transport (*SLC7A13*), protein glycosylation (*ALG1L*), and the olfactory receptor gene family (*OR2G2*) were decreased, whereas genes involved in immune response (*TNF, CCL4, CCL4L2, SERPINA2, ASB12*), cell stress (*NPPC*), and hedgehog signaling (*HHIP*) were increased.

The 7 coding genes that were differentially expressed following both low- and high-frequency tensile strain (vs. control) are involved in cytoskeleton organization, microtubule organization, cellular component assembly, and nucleotide-excision repair (*CETN1, DIRAS3, EVA1A, LRRN1, MYL1, TNNT3, XIRP1*) (Figure 1B).

*
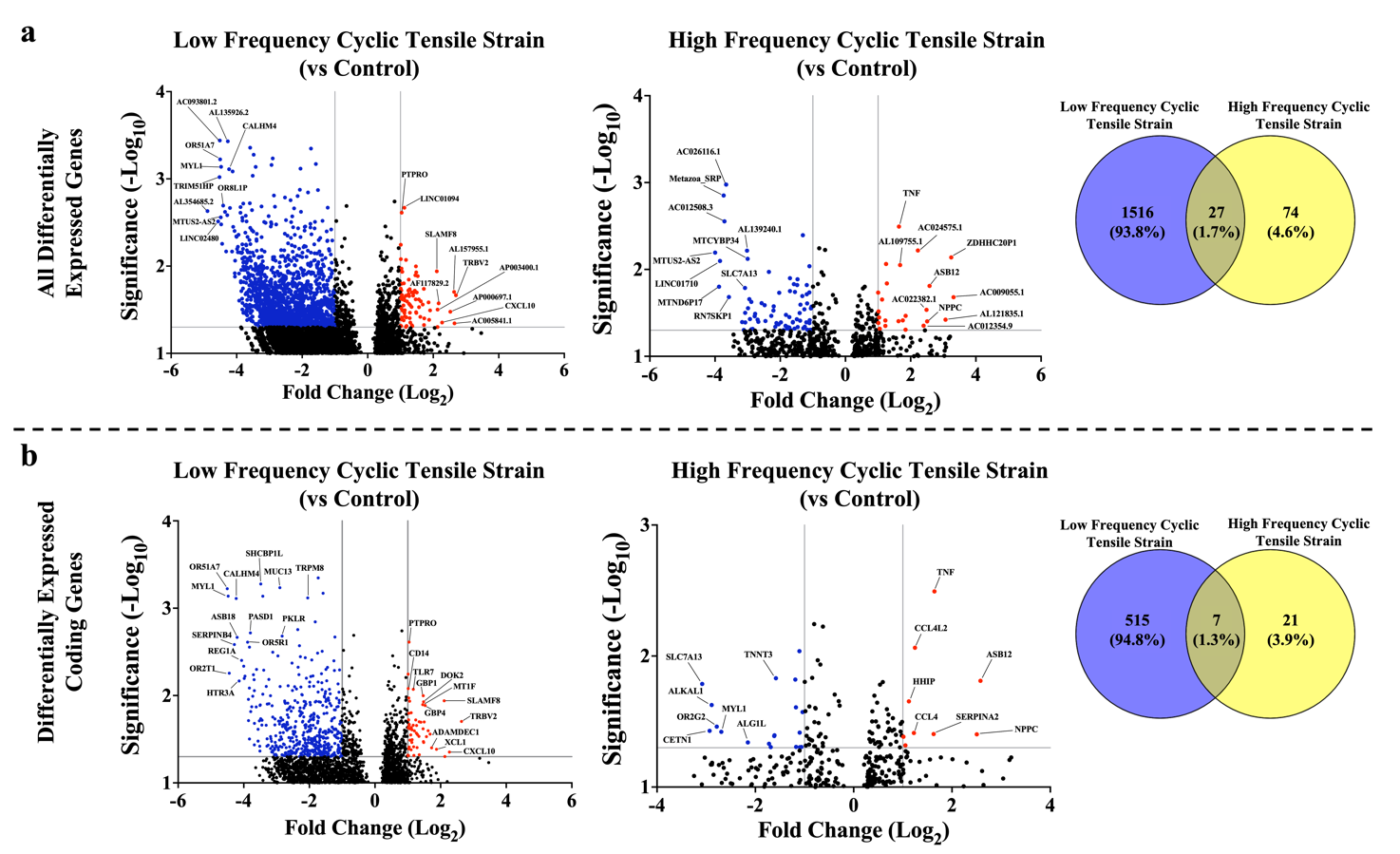
*

**Figure 1. Differentially expressed genes in Low- and High-frequency tensile strain conditions (versus Control). (A)** All differentially expressed genes and **(B)** differentially expressed coding genes. Each panel contains volcano plots displaying the top differentially expressed genes for low frequency cyclic tensile strain (vs control) and high frequency cyclic tensile strain (vs control). The Y-axis represents the -log10 p-value cut-off set at 1.3 (p-value < 0.05) and the X-axis represents the log2 fold change cut-off of below -1.0 (blue) or above 1.0 (red). Venn diagrams display the number of differentially expressed genes in common and unique to Low and High-frequency tensile strain conditions.

*Gene set enrichment analysis*

*Hallmark gene sets*: The top 10 significantly enriched Hallmark gene sets following exposure of OA synovial tissue to low-frequency, or high-frequency tensile strain (vs. control) are shown in Table 2. Forty-three gene sets were enriched by low-frequency tensile strain, including the same 26 gene sets significantly enriched by high-frequency tensile strain. Overall, cyclic tensile strain led to enrichment in gene sets related to inflammation (including TNF-α signaling via NFκB, interferon gamma and alpha responses), metabolism (including adipogenesis, glycolysis, oxidative phosphorylation), and cell stress (including hypoxia, apoptosis, and p53 pathway) (Supplementary File 1). Interestingly, high-frequency tensile strain primarily led to enrichment in cell metabolism (fatty acid metabolism, adipogenesis, and xenobiotic metabolism) with nominally higher normalized enrichment scores relative to low-frequency tensile strain (Table 2). We therefore conducted a more detailed analysis of cellular metabolism gene sets using Reactome.

| **Table 2**  **Top 10 Enriched gene sets from Hallmark for low and high frequency tensile strain** | | |
| --- | --- | --- |
| **HALLMARK: Low-frequency strain versus control** | | |
| **Gene Set** | **NES** | **FDR q-val** |
| Interferon Gamma Response | 4.18 | <0.0001 |
| Interferon Alpha Response | 4.00 | <0.0001 |
| Protein Secretion | 3.62 | <0.0001 |
| Oxidative Phosphorylation | 3.49 | <0.0001 |
| Allograft Rejection | 3.27 | <0.0001 |
| TNF-α Signaling via NFκB | 3.19 | <0.0001 |
| Apoptosis | 3.09 | <0.0001 |
| Inflammatory Response | 3.02 | <0.0001 |
| Adipogenesis | 2.93 | <0.0001 |
| Hypoxia | 2.82 | <0.0001 |
| **HALLMARK: High-frequency strain versus control** | | |
| **Gene Set** | **NES** | **FDR q-val** |
| Protein Secretion | 2.55 | <0.0001 |
| Oxidative Phosphorylation | 2.27 | <0.0001 |
| Inflammatory Response | 2.24 | <0.0001 |
| TNF-α Signaling via NFκB | 2.19 | <0.0001 |
| Allograft Rejection | 2.09 | <0.0001 |
| Hypoxia | 2.05 | <0.0001 |
| Fatty Acid Metabolism | 2.03 | <0.0001 |
| Apoptosis | 2.01 | <0.0001 |
| Adipogenesis | 1.96 | <0.0001 |
| Xenobiotic Metabolism | 1.88 | 0.001 |
| FDR, false discovery rate; NES, normalized enrichment score | | |

*Reactome gene sets*: Compared to controls, low-frequency tensile strain led to significant positive enrichment of 493 gene sets, while high-frequency tensile strain significantly enriched 97 gene sets. Low-frequency tensile strain induced enrichment of immune activation processes (*e.g.,* Fc receptor signalling pathways), while high-frequency tensile strain induced enrichment of more damaging immune responses (*e.g.,* NOD-signalling, neutrophil degranulation, NFκB translocation to nucleus, and NLR signaling pathways). There were 5 gene sets negatively enriched following low-frequency tensile strain related to cellular organization (keratinization, formation of cornified envelope), the mucin gene family, metabolism of amine-derived hormones, and olfactory signaling pathway, whereas no gene sets were negatively enriched in high-frequency tensile strain. Several significantly enriched gene sets related to cell metabolism and redox biology were exclusive to high-frequency tensile strain (Table 3). These included glyoxylate metabolism and glycine degradation, metabolic disorders of biological oxidation enzymes, macroautophagy, metabolism of fatty acids, citric acid cycle, diseases of metabolism, cytochrome P450, and others related to high levels of oxidative stress. The full lists of enriched Reactome gene sets can be found in Supplementary File 2.

| **Table 3**  **Metabolism-related Gene sets from Reactome exclusively enriched following high-frequency tensile strain** | | |
| --- | --- | --- |
| **REACTOME: Exclusive to high-frequency strain** | | |
| **Gene Set** | **NES** | **FDR q-value** |
| Glyoxylate metabolism and glycine degradation | 2.02 | 0.01 |
| Metabolic Disorders of biological oxidation enzymes | 2.00 | 0.01 |
| Cytochrome P450 Arranged by Substrate Type | 1.98 | 0.01 |
| Respiratory ETC ATP Synthesis by Chemiosmotic  Coupling and Heat Production by Uncoupling  Proteins | 1.83 | 0.03 |
| Macroautophagy | 1.82 | 0.03 |
| Fatty Acids | 1.76 | 0.03 |
| Citric Acid Cycle/TCA Cycle | 1.75 | 0.03 |
| ER Quality Control Compartment ERQC | 1.75 | 0.03 |
| Diseases of Metabolism | 1.73 | 0.04 |
| Endogenous Sterols | 1.69 | 0.04 |
| FDR, false discovery rate; NES, normalized enrichment score | | |

*Leading edge analysis*

Leading edge analysis of significantly enriched cell metabolism and cell stress gene sets identified different top genes involved in enrichment following high- and low-frequency strain (Table 4). Following exposure to high-frequency tensile strain (vs control) identified antioxidant genes for handling iron, superoxide, and nitric oxide (*FDX, DLD, SOD2*), AMPK-related genes (*PRKAB2, PRKAG2, PRKAA2*) responsible for regulating energy homeostasis and cell survival, genes related to galactose metabolism (*PGM2L1*) and reduction of P450 enzymes (*FDX*). Leading edge analysis of significantly enriched gene sets following exposure to low-frequency tensile strain (vs control) identified genes related to lipid metabolism and T-cell activation (HEXA, HEXB), lysosomal routing, stability, and activation (*GLB1*, *CTSA*^25^), and stimulation of proteasomal activity and/or mitophagy (*UBB, UBA52, UBC*). In tissues exposed to high-frequency strain (vs low-frequency) leading-edge identified *CYP* and *UGT* genes, which are activated by excess metabolite build up and by-products of severe oxidation^26-28^. While following low-frequency strain (vs high-frequency) leading edge genes included *RPS27A*, ubiquitin genes (*UBB, UBA52, UBC*), *MLST8*, and *ATP5F1B*, which indicate stimulation of protein turnover and mitophagy^29,30^.

| **Table 4**  **Top Reactome leading edge genes related to cell stress, ROS, NOS, and cell metabolism gene sets enriched by low- or high-frequency (vs. control), or low- vs high-frequency tensile strain.** | | | |
| --- | --- | --- | --- |
| **Low vs Control** | **High vs Control** | **Low vs High** | **High vs Low** |
| UBC | CYC5 | RPS27A | CYP1A2 |
| UBB | SOD2 | UBB | CYP2A13 |
| GLB1 | FDXR | UBA52 | CYP4F22 |
| RPS27A | PRKAB2 | UBC | CYP2J2 |
| CTSA | FDX1 | DLD | UGT1A1 |
| UBA52 | FDX2 | SDHC | UGT1A4 |
| GYG1 | PRKAG2 | SDHD |  |
| PRKAB2 | PGM2L1 | SDHA |  |
| HEXB | DLD | MLST8 |  |
| HEXA | PRKAA2 | ATP5F1B |  |

**Changes in lactate and cytokine/chemokine release into conditioned media following cyclic tensile strain**

Since gene sets associated with metabolic disturbances and oxidative stress were enriched following cyclic tensile strain, we compared lactate and cytokine/chemokine release from synovial tissues in conditioned media after tensile strain loading (Figure 2). Cyclic tensile strain increased lactate production (Figure 2A). High-frequency strain increased synovial tissue lactate release (442.11 nmol [95%CI 73.15, 811.06]) compared to static controls (Figure 2C). A small, statistically non-significant increase in lactate release was induced by low-frequency tensile strain (79.56 nmol [95%CI -289.40, 448.51]) compared to static control (Figure 2C).

MCP-1 concentration was highest in the conditioned media from control tissues (Figure 2B). Tissues exposed to low-frequency strain had decreased MCP-1 release (-3075.01 pg/mL [95%CI -5201.69, -948.33]) compared to control (Figure 2C). A similar trend was seen with tissues exposed to high-frequency strain (-2059.60 [95%CI -4186.28, 67.08]) compared to control (Figure 2C); however, this estimate lacked precision.


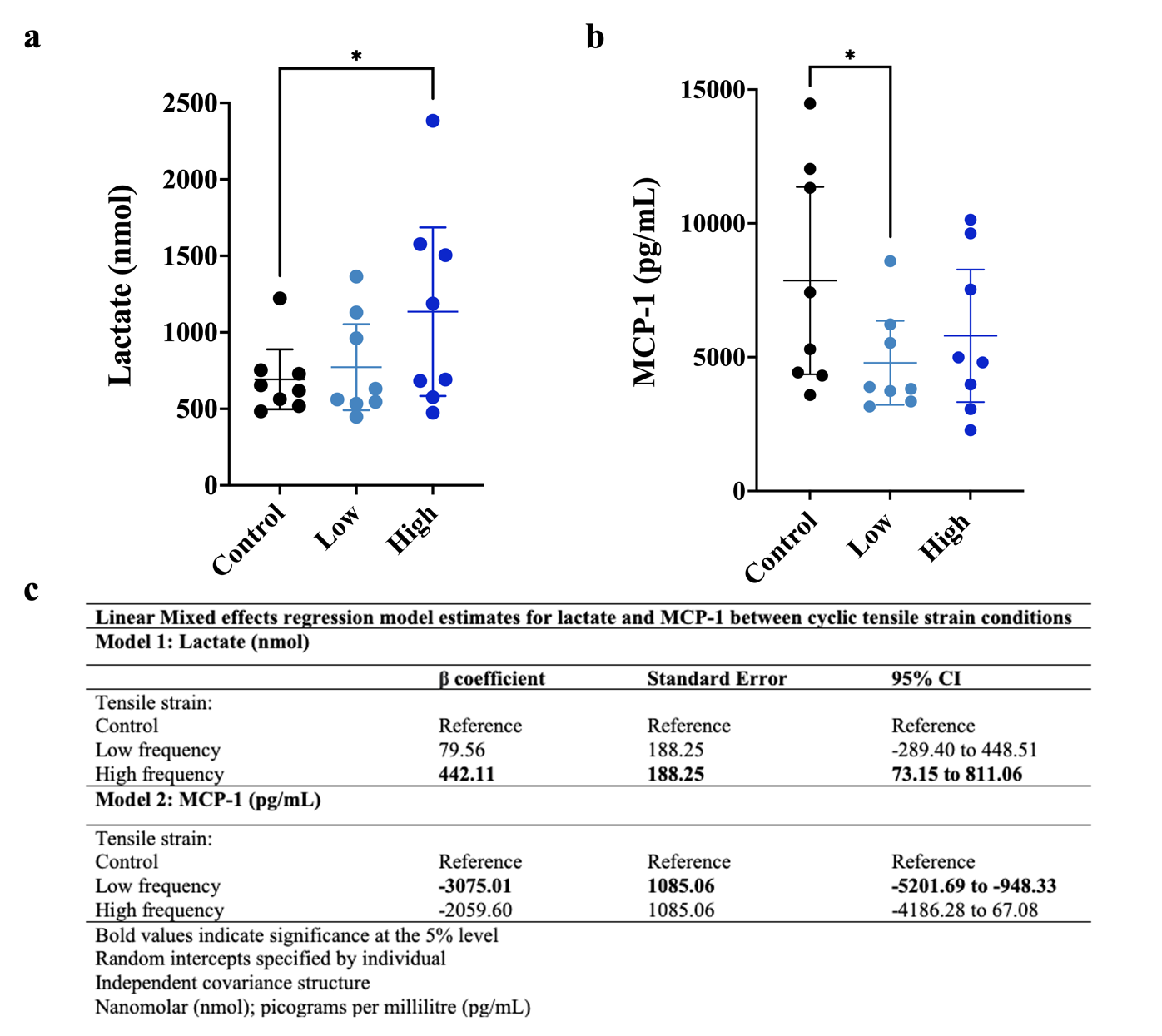


**Figure 2. Lactate and MCP-1 release into conditioned media from synovial tissue. (A)** Dot plot displaying the individual lactate measures as well as the mean ± 95% CI of lactate for Control (black), Low (light blue), and High (dark blue) strain conditions. **(B)** Dot plot displaying the individual MCP-1 measures as well as the mean ± 95% CI of lactate for Control (black), Low (light blue), and High (dark blue) strain conditions. **(C)** Linear mixed effects model estimates for lactate (Model 1) and MCP-1 (Model 2) in conditioned media for each cyclic tensile strain condition. CI, confidence interval; nmol, nanomolar; pg/mL, picogram per millilitre.

**Mechanical strain induces oxidation in synovial tissue**

Since our transcriptomics analysis identified strain-induced enrichment of numerous gene sets associated with oxidative stress, we measured 3-NT formation in synovial tissues (Figure 3). 3-NT increases under oxidative stress because of increased reactions between superoxide and nitric oxide and is an indicator of excess superoxide production and inflammation-associated increases in nitric oxide. Static control OA synovial tissues demonstrated a small amount of 3-NT staining in 6 out of 10 patients. High-frequency tensile strain led to an increase in percent 3-NT positive synovial intima lining cells (16.83 [95%CI 1.87, 31.79] compared to static control, while there was no clear change in 3-NT positive intimal cells following exposure to low-frequency strain (0.41 [95%CI -14.55, 15.37]) (Figure 3B) (Supplementary Table 1; Model 1). However, neither low- nor high-frequency strain led to an increase in percent 3-NT positive intimal lining CD68+ macrophages (Supplementary Table 1; Model 2). Interestingly, when controlling for the interaction between CD68+ macrophage content in the synovial intima and tensile strain frequency, the total %3-NT positive intimal lining cells increased even further (26.29 [95%CI 6.68, 45.89]) (Supplementary Table 1; Model 3).


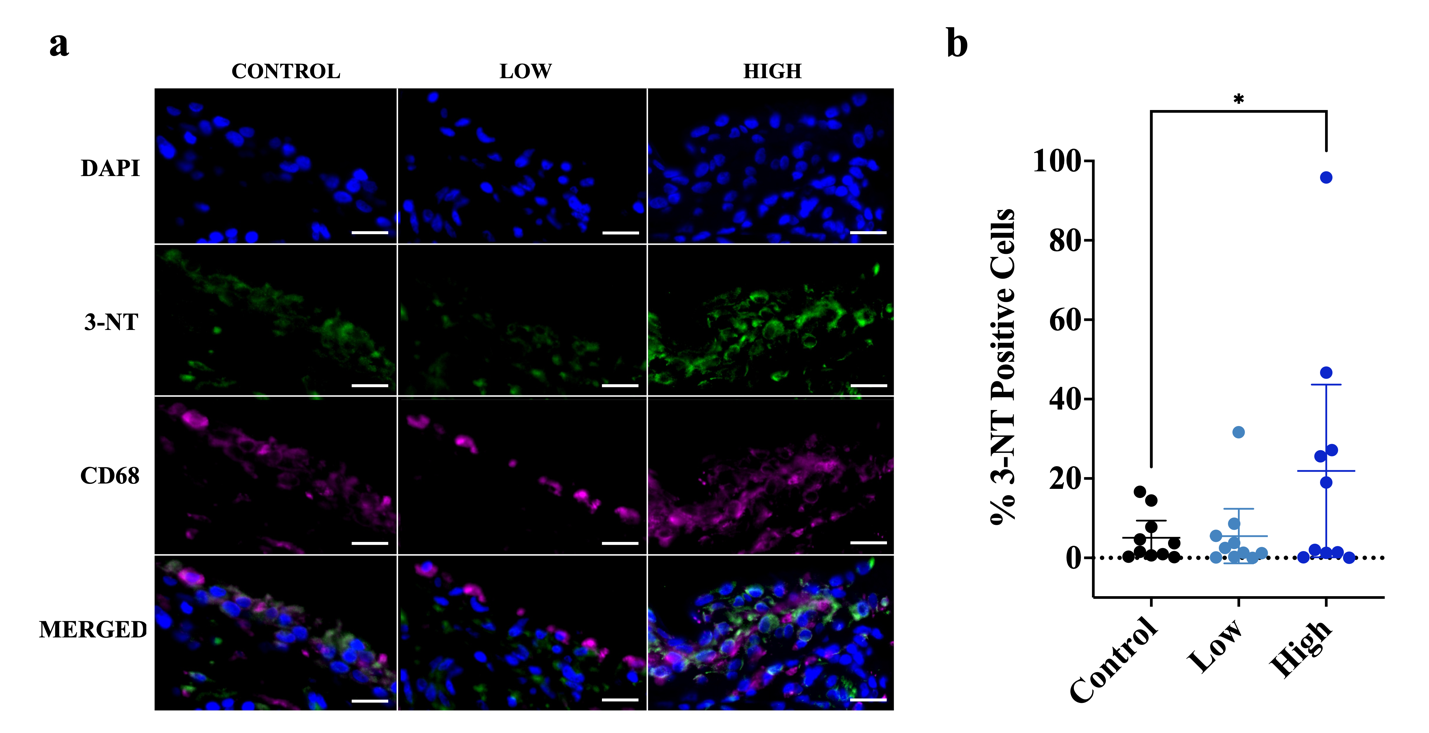


**Figure 3. Cyclic tensile strain increases 3-NT staining in OA synovial tissue. (A)** Representative immunofluorescence images of 3-NT (green) and CD68 (magenta) for each cyclic tensile strain loading condition. **(B)** Dot plots displaying the individual 3-NT+ cells (mean ± 95% CI) for Control (black), Low (light blue), and High (dark blue) tensile strain conditions. Scale bar = 20 μm. 3-NT, 3-Nitrotyrosine; CD68, macrophage marker; CI, confidence interval; DAPI, nuclear stain.

**DISCUSSION**

  The synovium is critical for maintaining joint health^3,4^ and synovial pathology (especially synovitis) is associated with pain and disease progression in OA. Characterizing synovial tissue responses to mechanical loading is therefore critical to understand the biological effects of physical activity, which is often associated with joint swelling and painful experiences for patients with knee OA. We found that intact synovial tissue demonstrated frequency-dependent responses to tensile strain. In particular, we observed changes in cell metabolism, oxidative stress, and inflammatory signaling.

Low-frequency tensile strain was better tolerated by OA synovium and led to enrichment in processes such as interferon-gamma and -alpha responses, Fc receptor signaling pathways, and lysosomal routing. Each of these processes are critical for recruitment of cells involved in immune regulation and resolution of innate immune responses^25,31,32^, suggesting that low-frequency strain may stimulate inflammation resolution mechanisms. This was further supported by decreased expression of *SERPIN* genes and the nuclear repressor of *CLOCK* (*PASD1*^33^). *SERPIN*s increase during inflammation to mitigate the damaging effects of uncontrolled enzyme activity and then decrease as inflammation resolves^34^. Similarly, CLOCK repressors inhibit the ability to maintain cell metabolism, homeostasis, and circadian rhythm^35,36^. Low-frequency tensile strain also led to enrichment of metabolism and cell stress pathways without inducing increases in lactate release or 3-NT formation, and reduced MCP-1 release compared to control. In contrast, high-frequency strain increased lactate release and 3-NT formation, abrogated the reduction in MCP-1 release, and stimulated greater enrichment of more harmful immune signaling processes such as NOD-like receptor signaling and neutrophil degranulation, suggesting a physiologic shift toward pro-inflammatory signaling pathways^37-39^. Similarly, glyoxylate metabolism was enriched, suggesting increases in ER stress and/or accumulation of glyoxylate in the cytoplasm causing cell damage^40^.

These whole-tissue findings are conceptually similar to an *in vitro* monolayer study by Takao *et al.* (2011), where tensile strain increased expression of pro-inflammatory genes (*COX2, IL-1β*) and synthesis of PGE2 in human synovial fibroblasts^19^. In that study, overexpression of hemeoxygenase-1 suppressed pro-inflammatory responses in fibroblasts, suggesting that the pro-inflammatory responses were due to mechanical induction of cellular oxidative stress^19^. Inflammation and oxidative stress are known to inhibit and damage mitochondria^41^, increasing levels of nitric oxide and superoxide and thus the formation of 3-NT. Our results therefore collectively suggest that the limits of OA synovial tissues to tolerate tensile strain forces were exceeded at high-frequency, but not low-frequency strain. Indeed, it is well-established that exercise and physical activity are beneficial for synovial joints, particularly in the medium to longer term^42,43^. However, patients with late-stage knee OA typically demonstrate acute, symptomatic intolerance to fast-paced walking, which aligns with our *ex vivo* observations in synovial tissue demonstrating that high-frequency tensile strain provokes cell stress responses in the short-term. Since we expect that these frequencies would be well-tolerated in healthy tissues, our findings suggest that OA may cause derangements in synovial tissue physiology and thereby lower tolerance to mechanical loads. This concept has been shown in the temporomandibular joint, where overloading shifts the homeostatic balance of anabolic and catabolic processes^44,45^. Such physiologic impairments may potentially have clinically relevant implications such as longer recovery times and/or poor joint and patient outcomes.

Our data provide some clues to how OA may lower tolerance to mechanical loading, resulting in inflammatory signaling and oxidative stress responses. High-frequency tensile strain led to higher energy demand with enrichment of glyoxylate metabolism and glycine catabolism, cytochrome metabolism, and ER stress pathways. These changes were driven in part by increased expression of genes responsible for handling excess metabolite build up and cell stress including *CYP, UGT*, and *FDX*^26-28^. Thus, OA may reduce the ability to process the buildup of excess metabolites during high energy demand, which would lead to ROS generation. Since synovial lining macrophages play important roles in OA inflammation and are an important source of reactive oxygen species (ROS) generation^46^, we were surprised that 3-NT staining was not increased in lining macrophages by tensile loading. Instead, higher proportions of synovial lining macrophages were associated with greater 3-NT staining in lining cells overall when we controlled for interactions between macrophage content and tensile strain frequency in our models. This suggests macrophages influence mechanobiological responses to tensile strain loading through nitric oxide signaling. Prior studies have also demonstrated that macrophages can modulate the redox metabolism of their resident tissue. A study by Wu *et al*. (2013) reported that cyclic strain activated inflammasome as a result of mitochondrial ROS in tissue-resident macrophages in the lung, which contributed to mechanical ventilation injury^47^. Considering our results, this concept should be investigated further in the context of OA. Additionally, since our findings are at the level of the whole tissue, future studies should consider single-cell RNA sequencing or spatial profiling to tease out the specific cell populations that may be responsible for the mechanobiological responses of the synovium.

Limitations of our study include the testing of synovial tissue samples only from late-stage OA patients undergoing joint replacement surgery, which may have responded differently from synovium from earlier stages of knee OA or healthy joints without OA. Future studies should attempt to include synovial biopsy samples from non-OA comparator tissues; however, access to these tissues in the quantity required for this type of experiment is often limited. Prior literature suggests that the forces experienced by synovial tissue in the mechanical stimulation protocol applied include both tensile strain and convective forces^1,2^, which should be considered when interpreting our findings. Mechanical stimulation occurred in atmospheric oxygen (considered hyperoxic compared to adapted cells in the body) and this may play a role in modulating some metabolic features of the response to tensile strain. We focused our measurements at 6-hours post-strain to assess early transcriptomic changes in response to loading, but different physiologic responses may occur immediately or much later after loading and should be explored in future studies. Patient confounders such as age, sex, BMI, and inflammatory status of the tissue may influence responses to cyclic tensile strain. We attempted to match patients by covariates, but these factors likely led to some degree of noise in our data, potentially precluding the detection of smaller physiologic responses to cyclic tensile strain.

Patients with OA experience increased pain or joint swelling during physical activity, which may in part be related to the mechanobiological responses of the synovium. To our knowledge, this is the first study to report transcriptomic and supporting physiologic data describing responses to tensile strain loading in whole synovial tissues from patients with knee OA. Given the strength of oxidative stress responses observed, we speculate that late-stage knee OA renders the synovium susceptible to oxidative stress during high frequency strain loading. Formation of 3NT implies the presence of high concentrations of nitric oxide and superoxide under strain conditions. Localization of these signals to the synovial lining and not macrophages themselves suggests that macrophages may be excreting nitric oxide that then interacts with intracellular superoxide in synoviocytes; however, this needs to be investigated further. Physiologic responses to tensile loading in late-stage OA synovium include increased inflammatory signaling mechanisms, shifts in cell metabolism, and redox-related cell stress, which appear to be frequency-dependent. These findings represent candidate mechanisms that may be relevant to activity-related joint outcomes and provide a useful experimental model for further investigation.

**METHODS**

**Study participants and synovial tissue biopsies**

Synovial tissue samples were collected from 10 sequential participants undergoing total knee arthroplasty (TKA) between January 2019 and July 2019. All included study participants had symptomatic, late-stage knee OA, with severe radiographic damage (Kellgren-Lawrence (KL) grade 3 or 4) warranting TKA. Patients provided written informed consent and the study was approved by Western University’s Research Ethics Board for Health Sciences Research Involving Human Patients (HSREB #109255) and this research was performed in accordance with the Declaration of Helsinki. During TKA, synovial biopsies were acquired from a standardized location in the lateral suprapatellar recess. An 8 mm diameter biopsy punch was used to collect standardized, adjacent biopsies of glassy-appearing synovial membrane, providing a set of 4 equal-sized pieces of tissue with a similar gross appearance from each patient. One piece of tissue was processed for routine histopathology with the remaining 3 pieces allocated to mechanical loading experiments.

**Cyclic tensile strain protocol**

A CellScale BioTester (CellScale, Waterloo, Canada) was used to apply controlled cyclic uniaxial strain to synovial tissue to model the tensile forces experienced by synovial tissues during rest and slow or fast flexion-extension joint movements. The tissue was secured to the BioTester using tissue Biorakes (tine diameter = 254 µm; tine spacing = 0.7 mm; puncture depth = 2.0 mm) and a 0.5 Newton (N) load cell was used for experimental tensile strain protocol. The fluid chamber was filled with DMEM and maintained at 37°C (Figure 4). Since there have been no previous studies assessing mechanical strain in whole synovium, limits testing was completed by loading pilot tissue samples at 10%, 20%, 30%, and 40% strain while monitoring force readouts for failure of the tissue (sudden drop in force), which occurred at 30% strain or higher. Ten percent strain was selected to apply gentle loading and avoid tissue failure and has been used previously in the literature for mechanical stimulation of cell monolayers^19^. Three matched pieces of synovial tissue per patient underwent one of 3 tensile strain (10%) loading protocols for 30 minutes each: 1) low-frequency cyclic tensile strain (0.3 Hz – 40 cycles/min), 2) high-frequency cyclic tensile strain (1.0 Hz –120 cycles/min), or 3) static control (tissues mounted on Biorakes and held for 30 minutes at 20 mN preload required to remain attached). Following each loading protocol, each tissue piece was placed in one well of a 12-well plate containing 1.0 mL of fresh serum-free DMEM, and incubated at 37°C, 5% CO_2_ for 6 hours. The conditioned media was then collected and placed in short-term cryostorage for downstream assays.


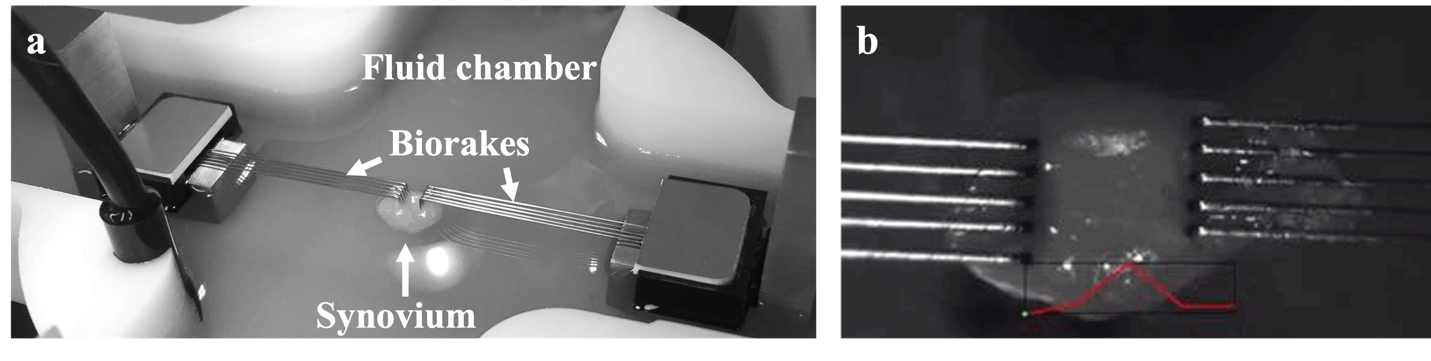


**Figure 4. Synovial cyclic tensile strain experimental set up. (A)** Representative image of synovial tissue sample loaded on CellScale BioTester apparatus. **(B)** Close up image of synovial tissue attached to Biorakes with strain displacement graph (red).

**Tissue homogenization and RNA purification**

Synovial tissues were bisected; one half was homogenized in 1mL of Trizol and stored at -20-degrees until RNA purification. The Directol-zol^TM^ RNA Microprep RNA purification kit (Zymo Research, Irvine, USA) was used to purify whole tissue RNA. The other half of the tissue was processed for paraffin embedding, histopathology, and immunostaining (see below).

**RNA library preparation and sequencing**

Total RNA was quantified using the NanoDrop (Thermo Fisher Scientific, Waltham, MA), RNA quality was assessed using the Agilent 2100 Bioanalyzer (Agilent Technologies Inc., Palo Alto, CA) and the RNA 6000 Nano kit (Caliper Life Sciences, Mountain View, CA). Synovial tissue RNA samples were included for RNA sequencing analysis if all 3 samples from an individual patient met the RNA quality (RIN >8.0) and quantity requirements. RNA sequencing was performed at the London Regional Genomics Centre (Robarts Research Institute, London, Canada). The TruSeq Stranded Total RNA Library Prep Gold kit (Illumina, San Diego, CA, USA) was used. Samples were rRNA depleted, fragmented, cDNA was synthesized, indexed, cleaned-up and amplified via PCR. Libraries were pooled in equimolar ratio into one library. Size distribution was assessed on an Agilent High Sensitivity DNA Bioanalyzer chip and quantitated using the Qubit 2.0 Fluorimeter (Thermo Fisher Scientific, Waltham, MA). The library was sequenced on an Illumina NextSeq 500 as 2x76bp paired end runs, using one High Output v2 kit (150 cycles).

**Analysis of RNA-sequencing data**

Raw FASTA files (runs and lanes) were concatenated to form the final FASTQ files to be used in the rest of the pipeline. R code was developed and run in RStudio (Version: 1.2.5001)^48^. fastp (version 0.20.0) was used to collect QC metrics of the raw reads^49^. RNA sequences were aligned and sorted by coordinates, to the NCBI human genome build 38 version 98, using STAR aligner (STAR-2.7.2c)^50^. The removal of alignment duplicates was done with Sambamba (version 0.7.0)^51^. Quantification of genes was performed using featureCounts (v1.6.3)^52^. DESeq2 (v 1.24.0)^53,54^ was used to normalize feature counts and to find the differentially expressed genes. The HGNC symbols were extracted and added to the DESeq2 results data frame using biomaRt (version 2.40.4)^55,56^ using the "hsapiens_gene_ensembl" dataset and the "Ensembl Release 98 (September 2019)" mart. Significantly differentially expressed genes were identified using the following criteria: p-value <0.05 (corresponds to a -log10[p-value] > 1.3) and a log2 fold change > ±1.0 (corresponds to an absolute fold change of 2.0). Volcano plots of differentially expressed genes were created using GraphPad Prism (version 9.0) software (San Diego CA, USA) with these criteria. Gene ontology (http://geneontology.org/) was used to determine pathway enrichment of the differentially expressed genes for each tensile strain condition: low-frequency versus control and high-frequency versus control. Venny 2.1.0^57^ was used to compare/contrast differentially expressed genes. Gene Set Enrichment Analysis (GSEA version 4.1.0)^58,59^ of normalized gene counts was used to identify significantly enriched gene sets/pathways by each tensile strain condition using the Hallmark and Reactome collections from the Molecular Signatures Database (MSigDB) as follows: low-frequency versus control, high-frequency versus control, and high-frequency versus low-frequency tensile strain. The Hallmark gene sets include 50 well-defined biological states or processes and the Reactome gene sets include over 1000 gene sets related to biological signaling, transcriptional regulation, and metabolism. Significant enrichment was determined by a false discovery rate (FDR) cut-off value of < 0.05. Leading edge analysis was completed in GSEA (version 4.1.0) to identify the top genes that contribute to the enrichment of multiple Reactome gene sets related to cell stress and cell metabolism.

**L(+)-lactate assay of conditioned media samples**

A fluorometric assay kit (Biovision, Mountain View, CA, USA) was used to measure L(+)-lactate release in conditioned media samples in duplicate using a microplate reader with excitation at 535 nm and emission at 590 nm. Amount (nanomolar; nmol) of lactate present in each sample was interpolated using the standard curve. Linear mixed effects regression models were used to assess differences in lactate between tensile strain loading conditions (control, low, and high), while adjusting for differences between patients (random intercepts). Results were reported as unstandardized beta (β) coefficients with 95% CIs and standard errors.

**Enzyme-linked immunosorbent assay of MCP-1 in conditioned media samples**

A LEGEND MAX^TM^ assay kit (BioLegend, San Diego, CA, USA) was used to measure monocyte chemoattractant protein-1 (MCP-1) in conditioned media samples in duplicate. Absorbance at 570 nm was subtracted from the absorbance reading at 450 nm. Amount (picogram per millilitre; pg/mL) of MCP-1 present in each sample was interpolated using the standard curve. Linear mixed effects regression models were used to assess differences in MCP-1 between tensile strain loading conditions (control, low, and high), while adjusting for differences between patients (random intercepts). Results were reported as unstandardized beta (β) coefficients with 95% CIs and standard errors.

**Synovial tissue histopathology**

Synovial tissue was fixed in 4% paraformaldehyde and paraffin embedded. Tissue samples were sectioned on a microtome at 5 µm thickness, stained with hematoxylin and eosin and scored using 5 non-overlapping, high-power fields (hpf, 400X) per patient. Six histopathological components were graded on a 0-3 scale (normal to severe) as previously described^60^. Mean and median scores for each component were calculated using the average or median score across 5 sections.

**Synovial tissue immunofluorescence**

Synovial tissues sections were deparaffinized and antigen retrieval was performed in sodium citrate buffer (10 mM sodium citrate, 0.05% Tween-20, pH 6) at 55°C overnight. Slides were permeabilized with Triton-X (0.2% in PBS; Sigma-Aldrich; 9002-93-1) and blocked for 1 hour with 5% bovine serum albumin (BSA; GE Healthcare). Samples were incubated with primary antibodies overnight at 4°C for anti-CD68 (Abcam, 1:200) in combination with anti-3-NT (Abcam, 1:150). Negative control sections without primary antibody were included to rule out non-specific secondary antibody binding. Slides were incubated with appropriate secondary antibodies including goat anti-mouse conjugated with Alexa Fluor-647 (Jackson ImmunoResearch Laboratories: 1:500) or goat anti-rabbit conjugated with Alexa Fluor-488 (Jackson ImmunoResearch Laboratories: 1:500). Coverslips were mounted using Molecular Probes ProLong Gold Antifade Mountant containing DAPI nuclear stain (Fisher Scientific, P36931). Slides were imaged at 40X using a Leica Aperio VERSA 8 microscopic scanner located at Molecular Pathology Core Facility, Robart’s Research Institute. Images were analyzed using QuPath (v0.2.3) cell detection and classifier tools^61^. We assessed 3-NT positive cells in the synovial intimal lining (normalized to cell density). We also assessed 3-NT staining relative to cell type content (*i.e.,* CD68+ (macrophage) vs CD68- cells in the synovial lining). Linear mixed effects regression was used to investigate 1) the association between percent positive 3-NT cells and tensile strain condition, while controlling for variation between patient samples (random intercepts) and 2) the association between 3-NT positive macrophages and tensile strain condition. Secondary analyses included 1) controlling for total synovial lining macrophage content and 2) including a strain by macrophage content interaction term. Results are reported as unstandardized beta coefficients (β) with 95% confidence intervals (CI).

**References:**

1 McCarty, W. J., Masuda, K. & Sah, R. L. Fluid movement and joint capsule strains due to flexion in rabbit knees. *J Biomech* **44**, 2761-2767 (2011). <https://doi.org:10.1016/j.jbiomech.2011.09.005> PMID - 21945567

2 Ingram, K. R., Wann, A. K. T., Angel, C. K., Coleman, P. J. & Levick, J. R. Cyclic movement stimulates hyaluronan secretion into the synovial cavity of rabbit joints. *J Physiology* **586**, 1715-1729 (2008). <https://doi.org:10.1113/jphysiol.2007.146753> PMID - 18202097

3 Mathiessen, A. & Conaghan, P. G. Synovitis in osteoarthritis: current understanding with therapeutic implications. *Arthritis Res Ther* **19**, 18 (2017). <https://doi.org:10.1186/s13075-017-1229-9> PMID - 28148295

4 Robinson, W. H. *et al.* Low-grade inflammation as a key mediator of the pathogenesis of osteoarthritis. *Nat Rev Rheumatol* **12**, 580-592 (2016). <https://doi.org:10.1038/nrrheum.2016.136> PMID - 27539668

5 Guermazi, A. *et al.* Synovitis in Knee Osteoarthritis Assessed by Contrast-enhanced Magnetic Resonance Imaging (MRI) is Associated with Radiographic Tibiofemoral Osteoarthritis and MRI-detected Widespread Cartilage Damage: The MOST Study. *J Rheumatology* **41**, 501-508 (2014). <https://doi.org:10.3899/jrheum.130541> PMID - 24429179

6 Hill, C. L. *et al.* Synovitis detected on magnetic resonance imaging and its relation to pain and cartilage loss in knee osteoarthritis. *Ann Rheum Dis* **66**, 1599 (2007). <https://doi.org:10.1136/ard.2006.067470> PMID - 17491096

7 Baker, K. *et al.* Relation of synovitis to knee pain using contrast-enhanced MRIs. *Ann Rheum Dis* **69**, 1779 (2010). <https://doi.org:10.1136/ard.2009.121426> PMID - 20472593

8 Oo, W. M. *et al.* Are OMERACT Knee Osteoarthritis Ultrasound Scores Associated With Pain Severity, Other Symptoms, and Radiographic and Magnetic Resonance Imaging Findings? *J Rheumatology* **48**, 270-278 (2020). <https://doi.org:10.3899/jrheum.191291> PMID - 32414954

9 Conaghan, P. G. *et al.* Clinical and ultrasonographic predictors of joint replacement for knee osteoarthritis: results from a large, 3-year, prospective EULAR study. *Ann Rheum Dis* **69**, 644 (2010). <https://doi.org:10.1136/ard.2008.099564> PMID - 19433410

10 Nielsen, F. K., Egund, N., Jørgensen, A. & Jurik, A. G. Risk factors for joint replacement in knee osteoarthritis; a 15-year follow-up study. *Bmc Musculoskelet Di* **18**, 510 (2017). <https://doi.org:10.1186/s12891-017-1871-z> PMID - 29202806

11 Coleman, M. C. *et al.* Targeting mitochondrial responses to intra-articular fracture to prevent posttraumatic osteoarthritis. *Sci Transl Med* **10**, eaan5372 (2018). <https://doi.org:10.1126/scitranslmed.aan5372> PMID - 29437147

12 Chou, C.-H. *et al.* Synovial cell cross-talk with cartilage plays a major role in the pathogenesis of osteoarthritis. *Sci Rep-uk* **10**, 10868 (2020). <https://doi.org:10.1038/s41598-020-67730-y> PMID - 32616761

13 Hunter, D. J. & Eckstein, F. Exercise and osteoarthritis. *J Anat* **214**, 197-207 (2009). <https://doi.org:10.1111/j.1469-7580.2008.01013.x> PMID - 19207981

14 Ernstgård, A., PirouziFard, M. & Thorstensson, C. A. Health enhancing physical activity in patients with hip or knee osteoarthritis - an observational intervention study. *Bmc Musculoskelet Di* **18**, 42 (2017). <https://doi.org:10.1186/s12891-017-1394-7> PMID - 28122519

15 Focht, B. C., Ewing, V., Gauvin, L. & Rejeski, W. J. The unique and transient impact of acute exercise on pain perception in older, overweight, or obese adults with knee osteoarthritis. *Ann Behav Med* **24**, 201-210 (2002). <https://doi.org:10.1207/s15324796abm2403_05> PMID - 12173677

16 Peeva, E. *et al.* A walking model to assess the onset of analgesia in osteoarthritis knee pain. *Osteoarthr Cartilage* **18**, 646-653 (2010). <https://doi.org:10.1016/j.joca.2009.12.008> PMID - 20175977

17 Sandal, L. F., Roos, E. M., Bøgesvang, S. J. & Thorlund, J. B. Pain trajectory and exercise-induced pain flares during 8 weeks of neuromuscular exercise in individuals with knee and hip pain. *Osteoarthr Cartilage* **24**, 589-592 (2016). <https://doi.org:10.1016/j.joca.2015.11.002> PMID - 26564575

18 Rohanifar, M. *et al.* Hydraulic Permeability and Compressive Properties of Porcine and Human Synovium. *Biophys J* (2022). <https://doi.org:10.1016/j.bpj.2022.01.008> PMID - 35032457

19 Takao, M. *et al.* Role of heme oxygenase-1 in inflammatory response induced by mechanical stretch in synovial cells. *Inflamm Res* **60**, 861-867 (2011). <https://doi.org:10.1007/s00011-011-0346-1> PMID - 21598088

20 Schröder, A. *et al.* Impact of Mechanical Load on the Expression Profile of Synovial Fibroblasts from Patients with and without Osteoarthritis. *Int J Mol Sci* **20**, 585 (2019). <https://doi.org:10.3390/ijms20030585> PMID - 30704030

21 Sambajon, V. V., Cillo, J. E., Gassner, R. J. & Buckley, M. J. The effects of mechanical strain on synovial fibroblasts. *J Oral Maxil Surg* **61**, 707-712 (2003). <https://doi.org:10.1053/joms.2003.50141> PMID - 12796883

22 Nazet, U. *et al.* Differential gene expression response of synovial fibroblasts from temporomandibular joints and knee joints to dynamic tensile stress. *J Orofac Orthop Fortschritte Der Kieferorthopädie*, 1-15 (2021). <https://doi.org:10.1007/s00056-021-00309-y> PMID - 34142176

23 Benito, M. J., Veale, D. J., FitzGerald, O., Berg, W. B. v. d. & Bresnihan, B. Synovial tissue inflammation in early and late osteoarthritis. *Ann Rheum Dis* **64**, 1263 (2005). <https://doi.org:10.1136/ard.2004.025270> PMID - 15731292

24 Wood, M. J. *et al.* Macrophage proliferation distinguishes 2 subgroups of knee osteoarthritis patients. *Jci Insight* **4**, e125325 (2019). <https://doi.org:10.1172/jci.insight.125325> PMID - 30674730

25 Timur, Z. K., Demir, S. A. & Seyrantepe, V. Lysosomal Cathepsin A Plays a Significant Role in the Processing of Endogenous Bioactive Peptides. *Frontiers Mol Biosci* **3**, 68 (2016). <https://doi.org:10.3389/fmolb.2016.00068> PMID - 27826550

26 Nebert, D. W., Wikvall, K. & Miller, W. L. Human cytochromes P450 in health and disease. *Philosophical Transactions Royal Soc B Biological Sci* **368**, 20120431 (2013). <https://doi.org:10.1098/rstb.2012.0431> PMID - 23297354

27 Sheftel, A. D. *et al.* Humans possess two mitochondrial ferredoxins, Fdx1 and Fdx2, with distinct roles in steroidogenesis, heme, and Fe/S cluster biosynthesis. *Proc National Acad Sci* **107**, 11775-11780 (2010). <https://doi.org:10.1073/pnas.1004250107> PMID - 20547883

28 Kalthoff, S., Ehmer, U., Freiberg, N., Manns, M. P. & Strassburg, C. P. Interaction between Oxidative Stress Sensor Nrf2 and Xenobiotic-activated Aryl Hydrocarbon Receptor in the Regulation of the Human Phase II Detoxifying UDP-glucuronosyltransferase 1A10*. *J Biol Chem* **285**, 5993-6002 (2010). <https://doi.org:10.1074/jbc.m109.075770> PMID - 20053997

29 Pickart, C. M. MECHANISMS UNDERLYING UBIQUITINATION. *Biochemistry-us* **70**, 503-533 (2001). <https://doi.org:10.1146/annurev.biochem.70.1.503> PMID - 11395416

30 Hochstrasser, M. Ubiquitin, proteasomes, and the regulation of intracellular protein degradation. *Curr Opin Cell Biol* **7**, 215-223 (1995). <https://doi.org:10.1016/0955-0674(95)80031-x> PMID - 7612274

31 Takai, T. Fc Receptors and Their Role in Immune Regulation and Autoimmunity. *J Clin Immunol* **25**, 1-18 (2005). <https://doi.org:10.1007/s10875-005-0353-8> PMID - 15742153

32 Lee, A. J. & Ashkar, A. A. The Dual Nature of Type I and Type II Interferons. *Front Immunol* **9**, 2061 (2018). <https://doi.org:10.3389/fimmu.2018.02061> PMID - 30254639

33 Michael, Alicia K. *et al.* Cancer/Testis Antigen PASD1 Silences the Circadian Clock. *Mol Cell* **58**, 743-754 (2015). <https://doi.org:10.1016/j.molcel.2015.03.031> PMID - 25936801

34 Bao, J. *et al.* Serpin functions in host-pathogen interactions. *Peerj* **6**, e4557 (2018). <https://doi.org:10.7717/peerj.4557> PMID - 29632742

35 Dibner, C., Schibler, U. & Albrecht, U. The Mammalian Circadian Timing System: Organization and Coordination of Central and Peripheral Clocks. *Annu Rev Physiol* **72**, 517-549 (2010). <https://doi.org:10.1146/annurev-physiol-021909-135821> PMID - 20148687

36 Gachon, F., Nagoshi, E., Brown, S. A., Ripperger, J. & Schibler, U. The mammalian circadian timing system: from gene expression to physiology. *Chromosoma* **113**, 103-112 (2004). <https://doi.org:10.1007/s00412-004-0296-2> PMID - 15338234

37 Wen, H., Miao, Edward A. & Ting, Jenny P. Y. Mechanisms of NOD-like Receptor-Associated Inflammasome Activation. *Immunity* **39**, 432-441 (2013). <https://doi.org:10.1016/j.immuni.2013.08.037> PMID - 24054327

38 Schroder, K., Kanneganti, T.-D., Shao, F. & Broz, P. Mechanisms and Consequences of Inflammasome Activation. *J Mol Biol* **430**, 131-132 (2018). <https://doi.org:10.1016/j.jmb.2017.12.005> PMID - 29247654

39 Wright, H. L., Moots, R. J. & Edwards, S. W. The multifactorial role of neutrophils in rheumatoid arthritis. *Nat Rev Rheumatol* **10**, 593-601 (2014). <https://doi.org:10.1038/nrrheum.2014.80> PMID - 24914698

40 Salido, E., Pey, A. L., Rodriguez, R. & Lorenzo, V. Primary hyperoxalurias: Disorders of glyoxylate detoxification. *Biochimica Et Biophysica Acta Bba - Mol Basis Dis* **1822**, 1453-1464 (2012). <https://doi.org:10.1016/j.bbadis.2012.03.004> PMID - 22446032

41 Chen, Y., Zhou, Z. & Min, W. Mitochondria, Oxidative Stress and Innate Immunity. *Front Physiol* **9**, 1487 (2018). <https://doi.org:10.3389/fphys.2018.01487> PMID - 30405440

42 Driban, J. B., Hootman, J. M., Sitler, M. R., Harris, K. & Cattano, N. M. Is Participation in Certain Sports Associated With Knee Osteoarthritis? A Systematic Review. *J Athl Training* **52**, 497-506 (2015). <https://doi.org:10.4085/1062-6050-50.2.08> PMID - 25574790

43 Lefèvre-Colau, M.-M. *et al.* Is physical activity, practiced as recommended for health benefit, a risk factor for osteoarthritis? *Ann Phys Rehabilitation Medicine* **59**, 196-206 (2016). <https://doi.org:10.1016/j.rehab.2016.02.007> PMID - 27103057

44 Asakawa-Tanne, Y. *et al.* Effects of Enzymatic Degradation after Loading in Temporomandibular Joint. *J Dent Res* **94**, 337-343 (2015). <https://doi.org:10.1177/0022034514560588> PMID - 25503611

45 Tanaka, E. & Koolstra, J. H. Biomechanics of the Temporomandibular Joint. *J Dent Res* **87**, 989-991 (2008). <https://doi.org:10.1177/154405910808701101> PMID - 18946004

46 Ryan, D. G. & O'Neill, L. A. J. Krebs Cycle Reborn in Macrophage Immunometabolism. *Annu Rev Immunol* **38**, 1-25 (2020). <https://doi.org:10.1146/annurev-immunol-081619-104850> PMID - 31986069

47 Wu, J. *et al.* Activation of NLRP3 Inflammasome in Alveolar Macrophages Contributes to Mechanical Stretch-Induced Lung Inflammation and Injury. *J Immunol* **190**, 3590-3599 (2013). <https://doi.org:10.4049/jimmunol.1200860> PMID - 23436933

48 Team, R. *RStudio: Integrated Develoopment for R.*, <<http://www.rstudio.com/>> (2020).

49 Chen, S., Zhou, Y., Chen, Y. & Gu, J. fastp: an ultra-fast all-in-one FASTQ preprocessor. *Bioinformatics* **34**, i884-i890 (2018). <https://doi.org:10.1093/bioinformatics/bty560> PMID - 30423086

50 Dobin, A. *et al.* STAR: ultrafast universal RNA-seq aligner. *Bioinformatics* **29**, 15-21 (2013). <https://doi.org:10.1093/bioinformatics/bts635> PMID - 23104886

51 Tarasov, A., Vilella, A. J., Cuppen, E., Nijman, I. J. & Prins, P. Sambamba: fast processing of NGS alignment formats. *Bioinformatics* **31**, 2032-2034 (2015). <https://doi.org:10.1093/bioinformatics/btv098> PMID - 25697820

52 Liao, Y., Smyth, G. K. & Shi, W. featureCounts: an efficient general purpose program for assigning sequence reads to genomic features. *Bioinformatics* **30**, 923-930 (2014). <https://doi.org:10.1093/bioinformatics/btt656> PMID - 24227677

53 Love, M. I., Huber, W. & Anders, S. Moderated estimation of fold change and dispersion for RNA-seq data with DESeq2. *Genome Biol* **15**, 550 (2014). <https://doi.org:10.1186/s13059-014-0550-8> PMID - 25516281

54 Team, R. C. *R: A language and environment for statistical computing.*, <<https://www.R-project.org/>> (2018).

55 Durinck, S. *et al.* BioMart and Bioconductor: a powerful link between biological databases and microarray data analysis. *Bioinformatics* **21**, 3439-3440 (2005). <https://doi.org:10.1093/bioinformatics/bti525> PMID - 16082012

56 Durinck, S., Spellman, P. T., Birney, E. & Huber, W. Mapping identifiers for the integration of genomic datasets with the R/Bioconductor package biomaRt. *Nat Protoc* **4**, 1184-1191 (2009). <https://doi.org:10.1038/nprot.2009.97> PMID - 19617889

57 Oliveros, J. VENNY. An interaction tool for comparing lists wiith Venn Diagrams.

58 Subramanian, A. *et al.* Gene set enrichment analysis: A knowledge-based approach for interpreting genome-wide expression profiles. *P Natl Acad Sci Usa* **102**, 15545-15550 (2005). <https://doi.org:10.1073/pnas.0506580102> PMID - 16199517

59 Mootha, V. K. *et al.* PGC-1α-responsive genes involved in oxidative phosphorylation are coordinately downregulated in human diabetes. *Nat Genet* **34**, 267-273 (2003). <https://doi.org:10.1038/ng1180> PMID - 12808457

60 Minten, M. J. M. *et al.* Exploring longitudinal associations of histologically assessed inflammation with symptoms and radiographic damage in knee osteoarthritis: combined results of three prospective cohort studies. *Osteoarthr Cartilage* **27**, 71-79 (2018). <https://doi.org:10.1016/j.joca.2018.10.014> PMID - 30448532

61 Bankhead, P. *et al.* QuPath: Open source software for digital pathology image analysis. *Sci Rep-uk* **7**, 16878 (2017). <https://doi.org:10.1038/s41598-017-17204-5> PMID - 29203879

**Acknowledgements**: The authors would like the acknowledge all members of the WOREO Knee Study team, especially Drs. Brent A. Lanting, Steven J. MacDonald, and Edward M. Vasarhelyi for access to synovial tissues. We would like to thank David Carter of the London Regional Genomics Centre (Robart’s Research Institute, London, ON, Canada) for library preparation and RNA sequencing, Hao Yin of the Molecular Pathology Core Facility (Robart’s Research Institute) for scanning our immunofluorescence slides, and Dale Fournier for his assistance with developing the BioTester cyclic tensile strain protocol.

**Author Contributions:**

All authors have made substantial contributions to the study conception and design, data acquisition, and data analysis and interpretation. All authors reviewed and approved the manuscript.

**Data Availability Statement:**

The RNA sequencing datasets generated and analyzed during the current study are available at Gene Expression Omnibus (GEO) repository, accession number: GSE205196. All other data is available upon reasonable request.

**Competing Interests:**

This work was funded in part by grants from the Academic Medical Organization of Southwestern Ontario (AMOSO), the Canada Research Chairs program, and Western University’s Bone and Joint Institute. HTP is supported by Frederick Banting and Charles Best Doctoral Award from CIHR. CTA is a consultant for Abbvie, Amgen, Bristol Myers Squibb, Celgene, Fresenius Kabi, Gilead, Janssen, Merck, Novartis, Pfizer, Hoffmaan LaRoche, Sandoz, Sanofi-Genzyme, and UCB. TBB, BF, LAW, MCC, and CAS declare no competing interests.

**Figure Legends:**

**Figure 1. Differentially expressed genes in Low- and High-frequency tensile strain conditions (versus Control). (A)** All differentially expressed genes and **(B)** differentially expressed coding genes. Each panel contains volcano plots displaying the top differentially expressed genes for low frequency cyclic tensile strain (vs control) and high frequency cyclic tensile strain (vs control). The Y-axis represents the -log10 p-value cut-off set at 1.3 (p-value < 0.05) and the X-axis represents the log2 fold change cut-off of below -1.0 (blue) or above 1.0 (red). Venn diagrams display the number of differentially expressed genes in common and unique to Low and High-frequency tensile strain conditions.

**Figure 2. Lactate and MCP-1 release into conditioned media from synovial tissue. (A)** Dot plot displaying the individual lactate measures as well as the mean ± 95% CI of lactate for Control (black), Low (light blue), and High (dark blue) strain conditions. **(B)** Dot plot displaying the individual MCP-1 measures as well as the mean ± 95% CI of lactate for Control (black), Low (light blue), and High (dark blue) strain conditions. **(C)** Linear mixed effects model estimates for lactate (Model 1) and MCP-1 (Model 2) in conditioned media for each cyclic tensile strain condition. CI, confidence interval; nmol, nanomolar; pg/mL, picogram per millilitre.

**Figure 3. Cyclic tensile strain increases 3-NT staining in OA synovial tissue. (A)** Representative immunofluorescence images of 3-NT (green) and CD68 (magenta) for each cyclic tensile strain loading condition. **(B)** Dot plots displaying the individual 3-NT+ cells (mean ± 95% CI) for Control (black), Low (light blue), and High (dark blue) tensile strain conditions. Scale bar = 20 μm. 3-NT, 3-Nitrotyrosine; CD68, macrophage marker; CI, confidence interval; DAPI, nuclear stain.

**Figure 4. Synovial cyclic tensile strain experimental set up. (A)** Representative image of synovial tissue sample loaded on CellScale BioTester apparatus. **(B)** Close up image of synovial tissue attached to Biorakes with strain displacement graph (red).
