# Supplementary material for: Tensile strain and altered synovial tissue metabolism in human knee osteoarthritis
Source: Sci Rep. 2022 Oct 17;12:17367. doi: 10.1038/s41598-022-22459-8 (PMC9576717; doi:10.1038/s41598-022-22459-8)
Supplement: Supplementary file 6 — Supplementary Table 1. [file 41598_2022_22459_MOESM6_ESM.pdf]

Supplementary Material

Supplementary Table 1
Linear mixed effects regression model estimates for 3-NT immunofluorescence between tensile strain conditions

	**β coefficient**	**Standard Error**	**95% CI**
**Model 1: %3-NT+ cells**			
Tensile strain:			
Control	Reference	Reference	Reference
Low frequency	0.41	7.63	-14.55 to 15.37
High frequency	**16.83**	**7.63**	**1.87 to 31.79**
**Model 2: %3-NT+ macrophages**			
Tensile strain:			
Control	Reference	Reference	Reference
Low frequency	-5.90	7.85	-21.29 to 9.49
High frequency	1.86	7.85	-13.52 to 17.25
**Model 3: %3NT+ cells with strain x Total lining macrophage interaction**			
Tensile strain:			
Control	Reference	Reference	Reference
Low frequency	-0.45	10.35	-20.74 to 19.84
High frequency	**26.29**	**10.00**	**6.68 to 45.89**
Total Lining Macrophages	0.27	0.36	-0.44 to 0.98
**Tensile Strain x Total Lining Macrophages**			
Control x Total lining macrophages	Reference	Reference	Reference
Low frequency x Total lining macrophages	0.14	0.56	-0.95 to 1.23
High frequency x Total lining macrophages	-0.81	0.53	-1.86 to 0.23

Bolded value indicates significance at the 5% level
Random intercepts specified by individual
Independent covariance structure
